# Supplementary material for: Precision dynamical mapping using topological data analysis reveals a hub-like transition state at rest
Source: Nat Commun. 2022 Aug 15;13:4791. doi: 10.1038/s41467-022-32381-2 (PMC9378660; doi:10.1038/s41467-022-32381-2)
Supplement: Supplementary file 1 — Supplementary Information [file 41467_2022_32381_MOESM1_ESM.pdf]

**Supplementary Information for:**

# Precision dynamical mapping using topological data analysis reveals a hub-like *transition state* at rest

Manish Saggar[a*], James M. Shine[b], Raphaël Liégeois[c,d], Nico U. F. Dosenbach[e], Damien Fair[f]

[a]Department of Psychiatry and Behavioral Sciences, Stanford University, Stanford, CA, USA
[b]Brain and Mind Center, The University of Sydney, Sydney, New South Wales, Australia
[c]Institute of Bioengineering, École Polytechnique Fédérale de Lausanne, Switzerland
[d]Department of Radiology and Medical Informatics, Faculty of Medicine, University of Geneva
[e]Departments of Neurology, Radiology, Pediatrics and Biomedical Engineering, Washington University School of Medicine, St. Louis, MO, USA
[f]Department of Pediatrics, University of Minnesota Medical School, Minneapolis, MN, USA

* Corresponding Author: saggar@stanford.edu

## Supplementary Figures

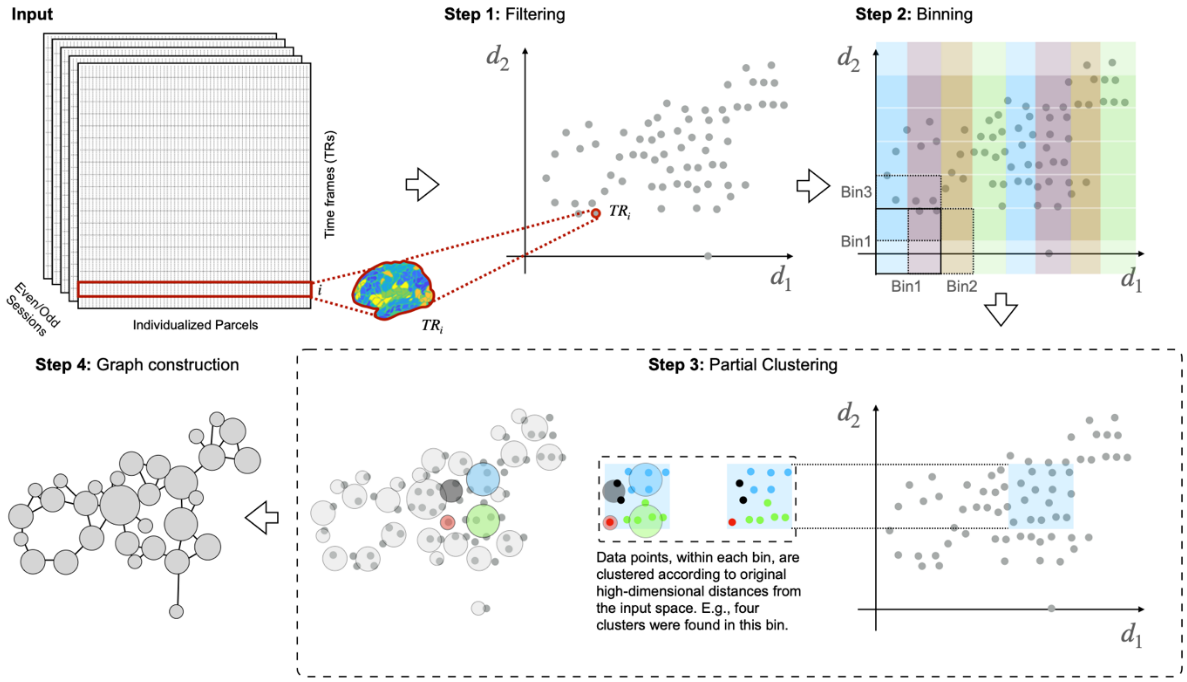


**Fig. S1: Step-by-step representation of the Mapper pipeline.** First, the high-dimensional neuroimaging data are embedded into a lower dimension set $d_i$, using a non-linear filter function $f$. Here, a nonlinear filter function $f$ based on neighborhood embedding was used (see Methods for benefits of this non-linear approach). Second, overlapping $d$-dimensional binning is performed to allow for compression and to reduce the destructive effects of noise. Third, partial clustering within each bin is performed, where the original high dimensional information is used for coalescing (or separating) data points into nodes in the low-dimensional space and hence allows for recovering information loss incurred due to dimensional reduction. As a fourth step, to generate a graphical representation of the data landscape, nodes from different bins are connected if any data points are shared between them.

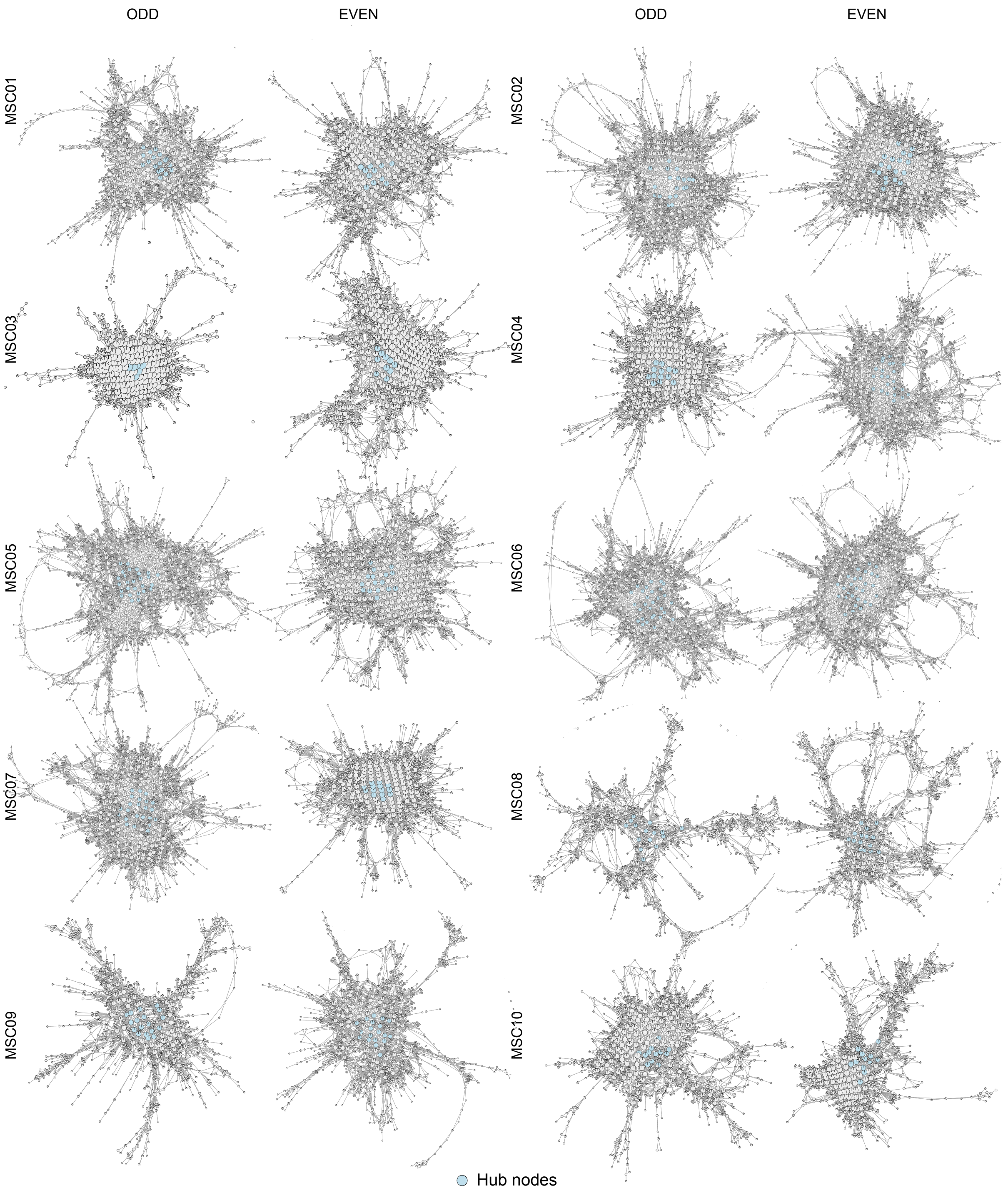


**Fig. S2: Shows hubs across all ten MSC participants.** Show Mapper-generated graphs for all 10 MSC participants and their respective data splits. Hubs (i.e., nodes with high degree (>20) and high centrality (top 1%)) are highlighted in blue color. As evident, these hubs were found across all participants and sessions.

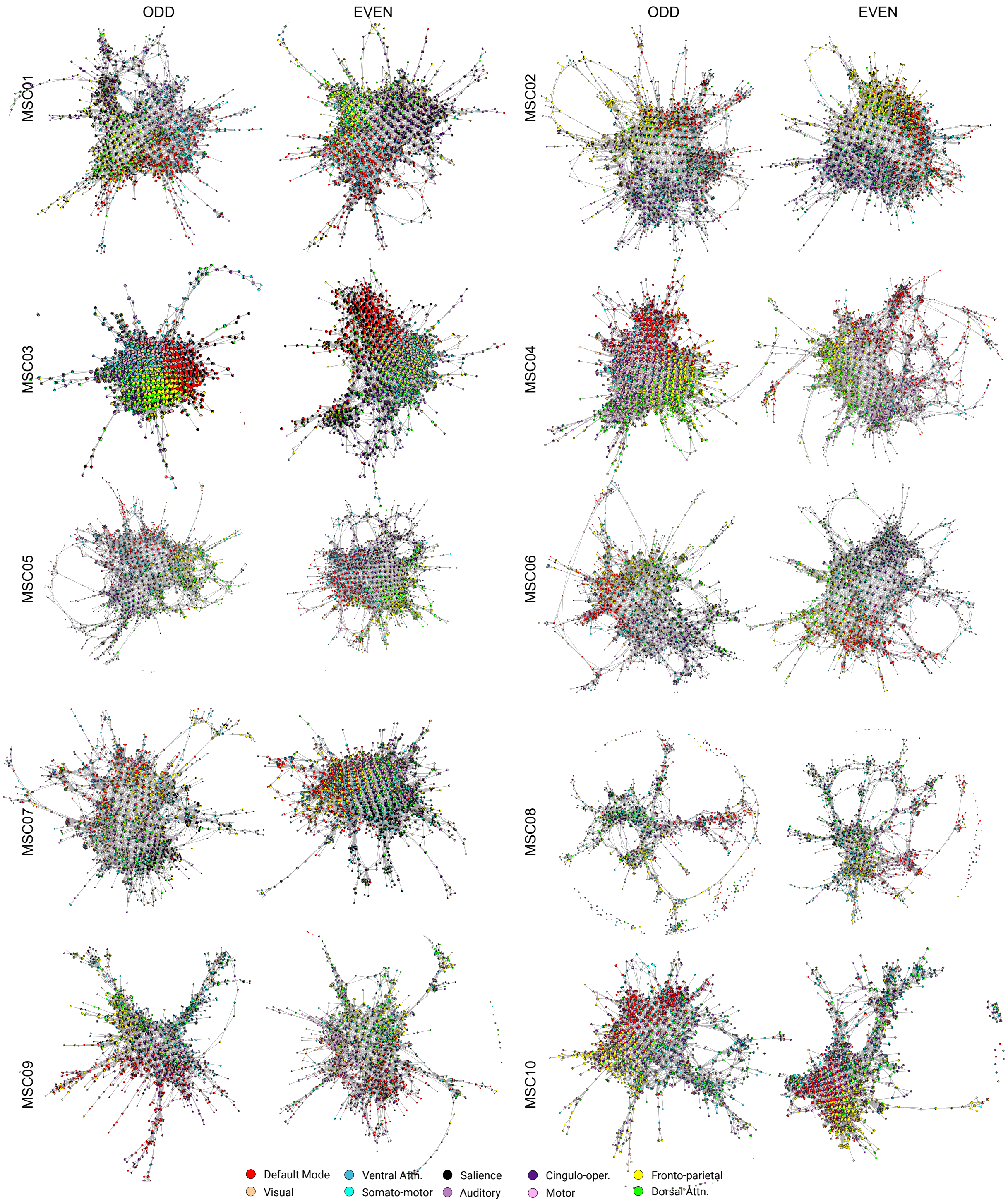


**Fig. S3: RSN-based annotation across all ten MSC participants.** Shows Mapper-generated graphs for all 10 MSC participants. Here, each node is annotated by activation in the known large-scale resting state networks. Each node is annotated using a pie-chart to show the proportion of RSNs activated within each node. As evident, topologically highly connected, and centrally located hubs contained brain volumes where no characteristic RSN was activated above the mean, whereas nodes with brain volumes dominating from one (or more) RSN(s) tend to occupy the peripheral corners of the landscape. The maps for all individual subjects

demonstrated this same basic pattern, although there was evidence to suggest that different combinations of RSNs were dominant in different individuals.

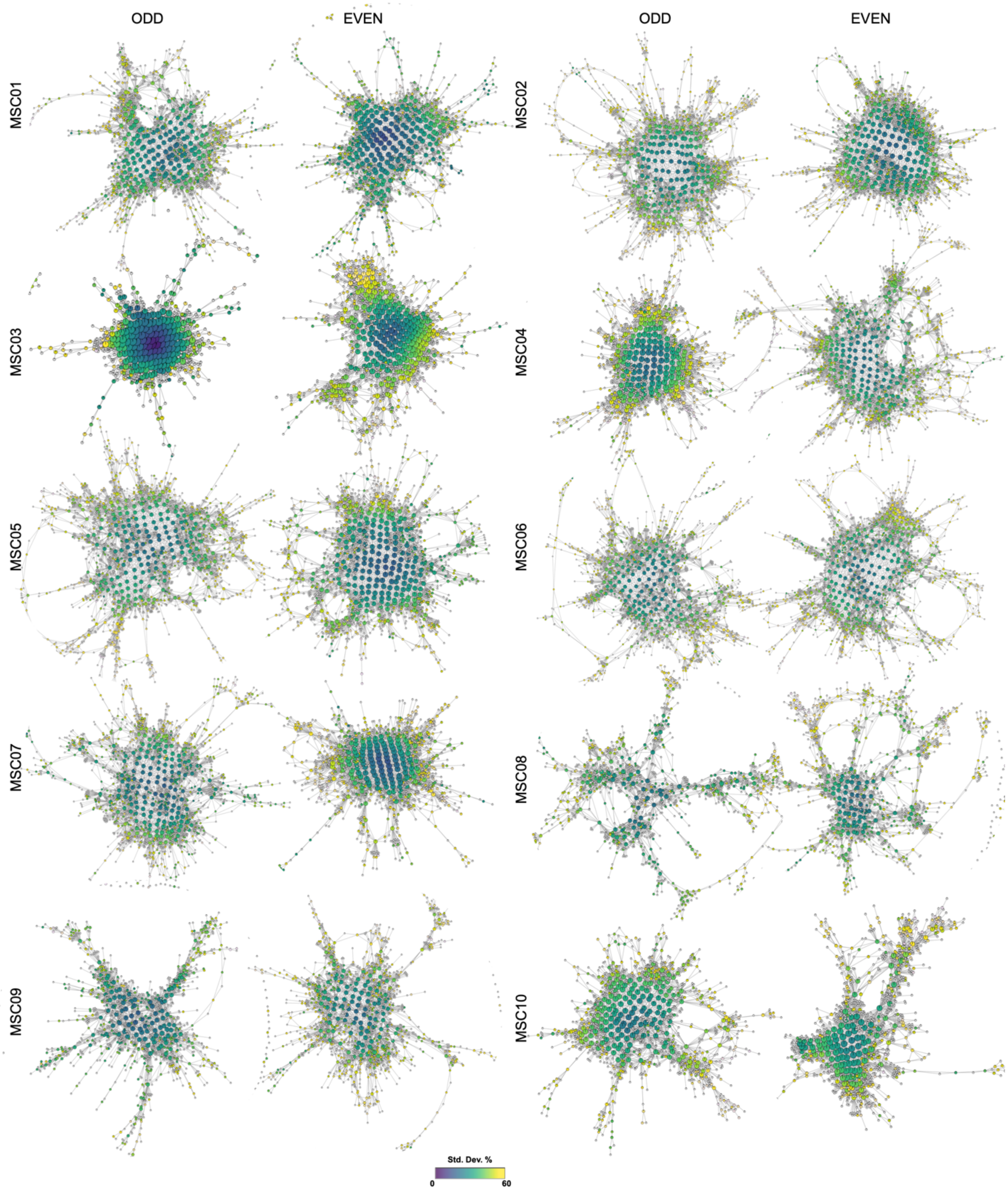


**Fig. S4: Gradient was observed across all ten MSC participants**. To quantify the variation in RSN-based dominance, we first estimated mean activation for each RSN across the time frames within each node, followed by estimating variation in mean activation across RSNs. High variance (or S.D.) indicated dominance of one or more RSN while low variance (or S.D.) indicated uniformity across RSN activation. Annotating Mapper-generated graphs using variance-based approach revealed a dynamical topographic gradient, where the peripheral

nodes had higher variance with a continual decrease in variance when going towards the center of the graph. This topographic gradient was observed across all participants and sessions.

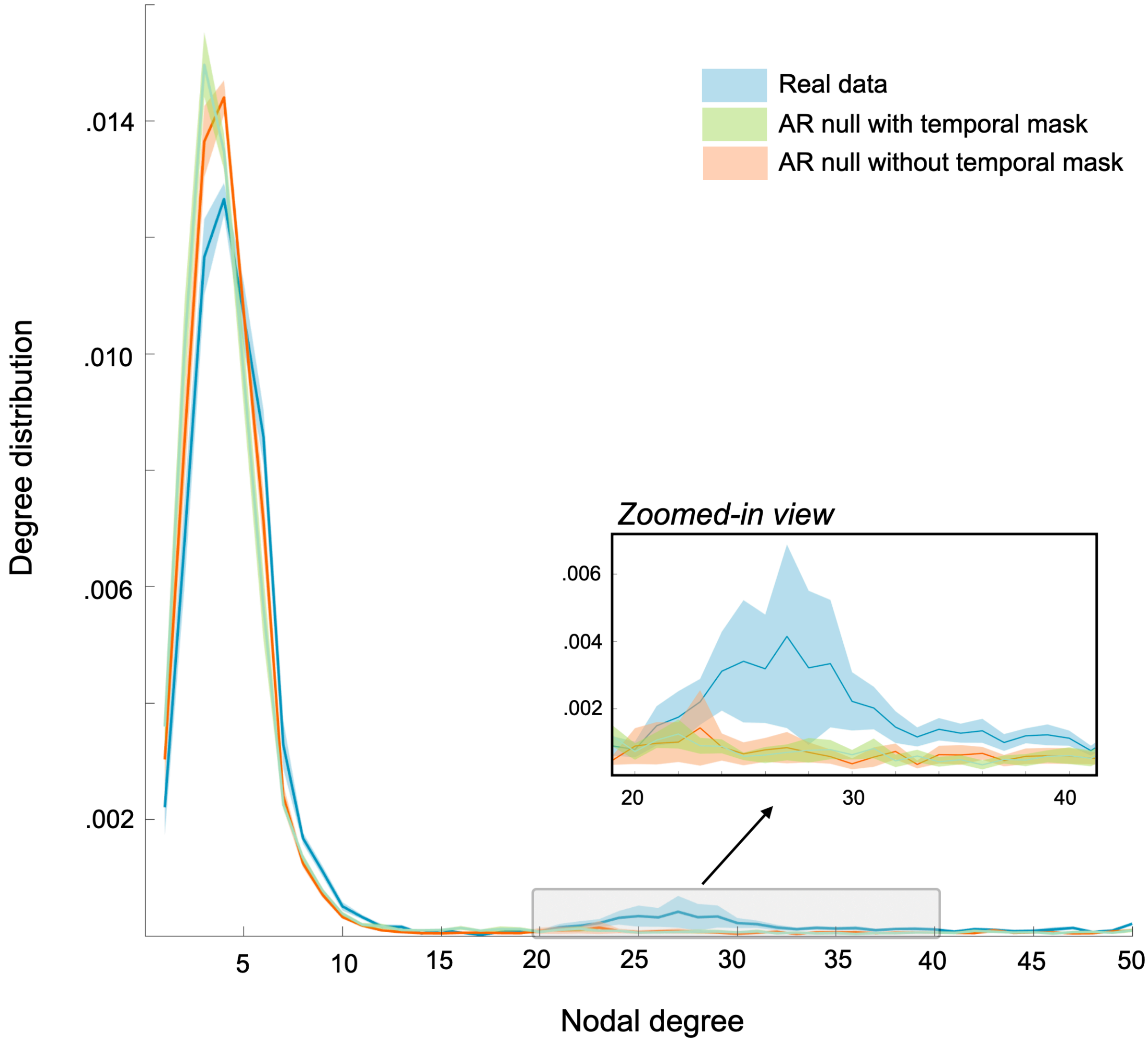


**Fig. S5: Applying frame censoring to the data generated from null models to evaluate whether high degree nodes observed in the real data were resulted merely due to temporal masking**. Please note that the frame censoring could only be applied to the data generated from the AR null, as it is a generative model, and we could create same number of TRs as the original (non-censored) data and then drop frames from it to match the number of frames in the real data. As evident, adding frame censoring to the data generated using AR null did not result in enhancing the number of high degree nodes. Statistically, the proportion of high degree nodes in real data were still significantly higher than both AR nulls (one-way ANOVA; $F=4.11$, $p=0.0276$). Only showing data from the odd split, similar results were observed for the even split of the data. The shaded area represents standard error around the mean (S.E.M.).

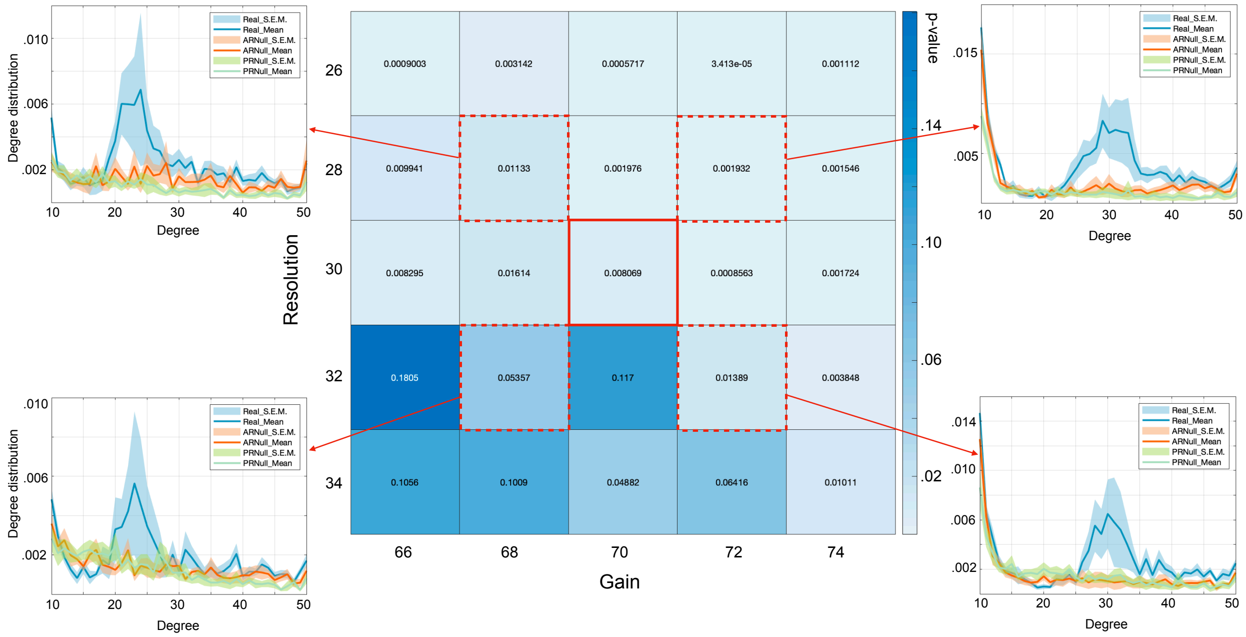


**Fig. S6: Parameter perturbation analysis revealed stable results across a moderate range of Mapper parameters.** Parameter perturbation analysis was performed to make sure topological properties of the graph (e.g., existence of fat tail in degree distribution) were stable across a moderate range of Mapper parameters. Two main Mapper parameters, i.e., number of bins (a.k.a. Resolution (R)) and percentage overlap between bins (a.k.a. Gain (g)) were varied across the chosen value in the main text (R=30, G=70). The values of R and G were chosen based on our previous work with task fMRI data (Saggar et al. 2018; Nat. Comm.). The heatmap above shows p-values from one-way ANOVAs that examined the proportion of high-degree nodes (>20) in the real versus null data for the odd sessions. As evident, for a large portion of Mapper parameter values, the proportion of high-degree nodes in the real data were significantly higher than null data. We also depict zoomed-in view of degree distribution plots for several parameter combinations (highlighted in red-dashed border on the heatmap) to show the excessive proportion of high-degree nodes in real data across different combinations of parameters. The shaded area in the degree distribution plots represents standard error around the mean (S.E.M.).

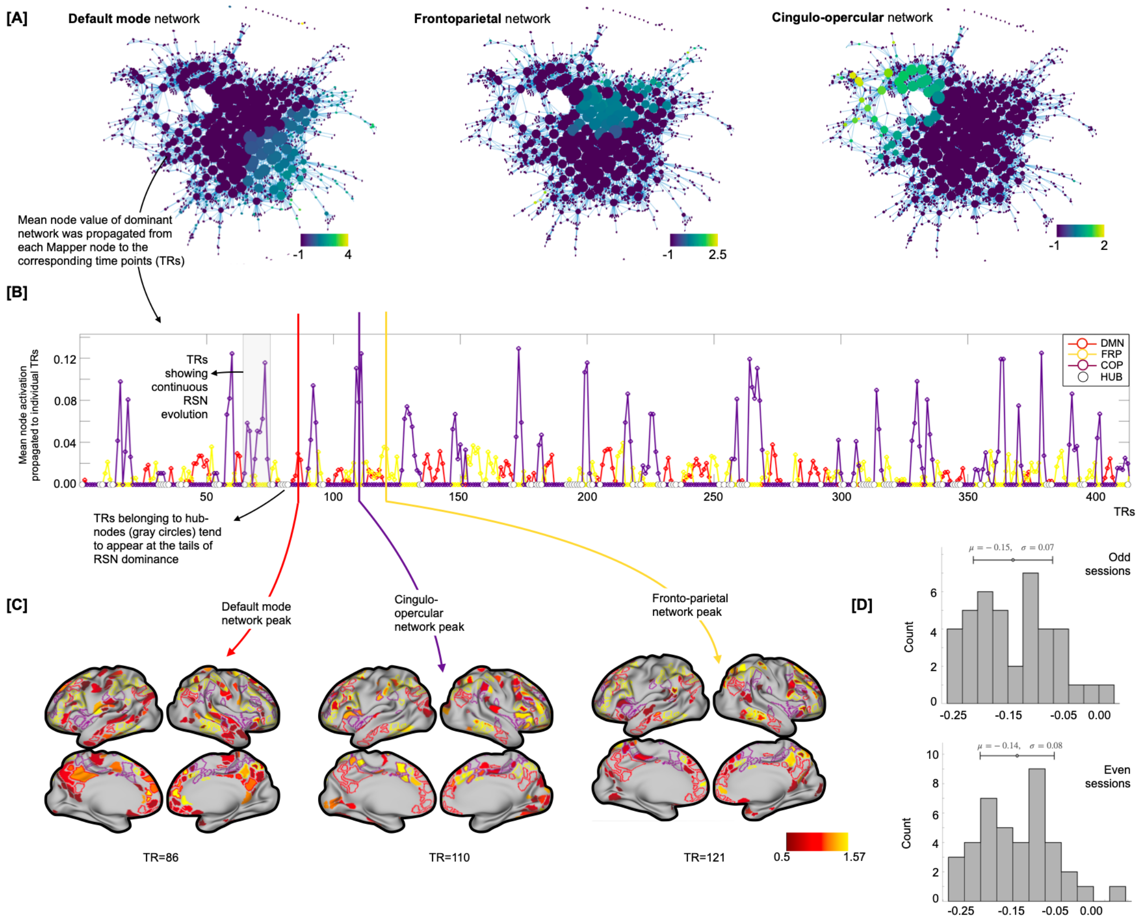


**Fig. S7**: **The nodal mean of each dominant network was propagated into time domain (individual TRs) to examine the continuous and transitory nature of RSN-dominance vs hub-states. [A]** Mapper nodes are annotated by activation in three dominant RSNs relative to mean activations of all other RSNs for one representative participant (MSC-01, odd sessions). As expected, for each dominant RSN, we observed a gradient of mean activation across nodes in the Mapper graph – such that peripheral nodes contained timeframes (or TRs) with the highest activation, while more central nodes contained TRs with low activation. As evident in **[B],** the activation of each of the three dominating networks (default mode, frontoparietal, and cingulo-opercular) are continuous in nature and, importantly, the hub-states tend to appear at the tails of RSN dominance – putatively triggering transitions between RSNs. **[C]** Cortical activations are shown for three representative TRs for each dominant network. **[D]** Histogram of temporal correlation between the mean amplitude of dominant RSNs and hub-state occurrences across all ten participants (separately shown for odd and even sessions). As evident, negative relation between the occurrence of hub-states and activation in one or more RSNs was observed. The brain overlays were created by the authors using Connectome Workbench Software (https://www.humanconnectome.org/software/connectome-workbench).

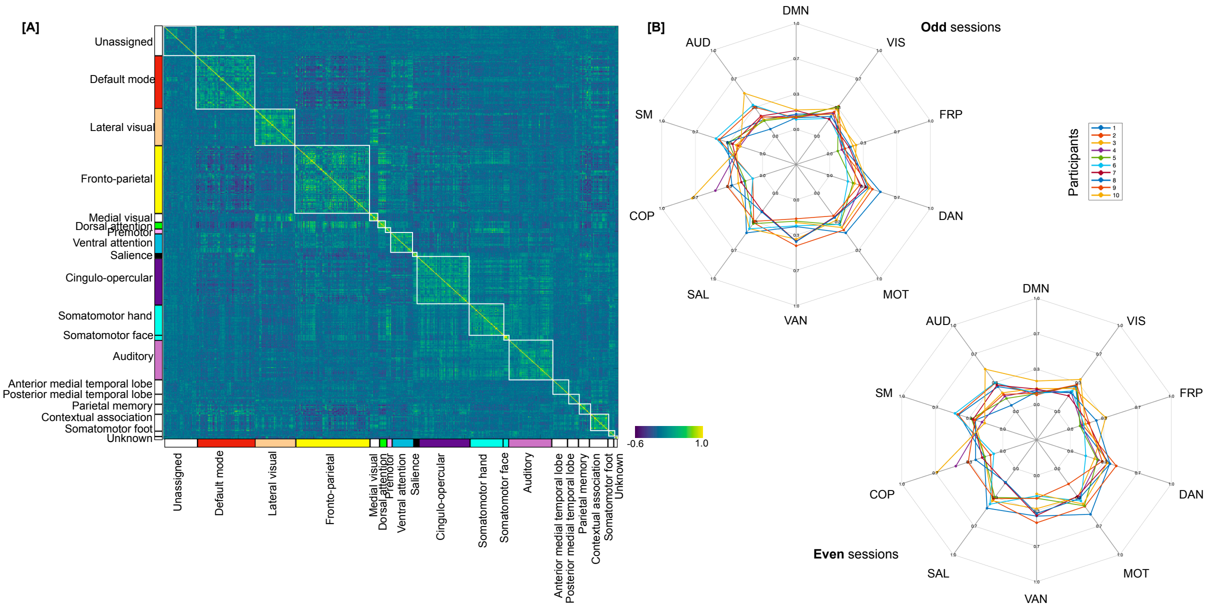


**Fig. S8: Examining connectivity profile of hub states.** [A] Functional connectivity derived from timeframes of hub Mapper nodes for one representative participant (MSC01, odd sessions). The connectivity matrix is organized by RSNs. Uniform within network connectivity is observed across all networks. [B] Spider charts showing within-network connectivity (derived from hubs) across all 10 participants, separately for odd and even sessions. Although Mapper graphs were generated using activation data (and not connectivity estimates), within network functional connectivity derived from hubs also suggest no preference for any RSN.
