# Supplementary material for: Precision dynamical mapping using topological data analysis reveals a hub-like transition state at rest
Source: Nat Commun. 2022 Aug 15;13:4791. doi: 10.1038/s41467-022-32381-2 (PMC9378660; doi:10.1038/s41467-022-32381-2)
Supplement: Supplementary file 3 — Reporting Summary [file 41467_2022_32381_MOESM3_ESM.pdf]

nature portfolio

Corresponding author(s): Manish Saggar

Last updated by author(s): May 26, 2022

Reporting Summary

Nature Portfolio wishes to improve the reproducibility of the work that we publish. This form provides structure for consistency and transparency in reporting. For further information on Nature Portfolio policies, see our Editorial Policies and the Editorial Policy Checklist.

Statistics

For all statistical analyses, confirm that the following items are present in the figure legend, table legend, main text, or Methods section.

n/a	Confirmed	
☐	☒	The exact sample size (*n*) for each experimental group/condition, given as a discrete number and unit of measurement
☐	☒	A statement on whether measurements were taken from distinct samples or whether the same sample was measured repeatedly
☐	☒	The statistical test(s) used AND whether they are one- or two-sided<br>*Only common tests should be described solely by name; describe more complex techniques in the Methods section.*
☐	☒	A description of all covariates tested
☒	☐	A description of any assumptions or corrections, such as tests of normality and adjustment for multiple comparisons
☐	☒	A full description of the statistical parameters including central tendency (e.g. means) or other basic estimates (e.g. regression coefficient) AND variation (e.g. standard deviation) or associated estimates of uncertainty (e.g. confidence intervals)
☐	☒	For null hypothesis testing, the test statistic (e.g. *F*, *t*, *r*) with confidence intervals, effect sizes, degrees of freedom and *P* value noted<br>*Give P values as exact values whenever suitable.*
☒	☐	For Bayesian analysis, information on the choice of priors and Markov chain Monte Carlo settings
☒	☐	For hierarchical and complex designs, identification of the appropriate level for tests and full reporting of outcomes
☐	☒	Estimates of effect sizes (e.g. Cohen's *d*, Pearson's *r*), indicating how they were calculated

Our web collection on statistics for biologists contains articles on many of the points above.

Software and code

Policy information about availability of computer code

Data collection	We used two already collected datasets for this work. (1) Midnight scan club (MSC) dataset: These data were obtained from the OpenNeuro database. Its accession number is ds000224. (2) Human Connectome Project (HCP) dataset, n=100 cohort; These data were directly obtained from the HCP website (db.humanconnectome.org)
Data analysis	We used fMRIPrep v1.5.9 to preprocess fMRI data. Mapper construction was done based on a Matlab-based toolbox, available here https://github.com/braindynamicslab/tda-msc-rsfMRI. For graph theoretical analysis, we used the Brain Connectivity Toolbox (https://sites.google.com/site/bctnet/). For analyzing fMRI data we used FSL toolbox v6.0 (available here https://fsl.fmrib.ox.ac.uk/fsl/fslwiki).

For manuscripts utilizing custom algorithms or software that are central to the research but not yet described in published literature, software must be made available to editors and reviewers. We strongly encourage code deposition in a community repository (e.g. GitHub). See the Nature Portfolio guidelines for submitting code & software for further information.

Data

Policy information about availability of data

All manuscripts must include a data availability statement. This statement should provide the following information, where applicable:

- Accession codes, unique identifiers, or web links for publicly available datasets
- A description of any restrictions on data availability
- For clinical datasets or third party data, please ensure that the statement adheres to our policy

We used two already collected datasets for this work. (1) Midnight scan club dataset: These data were obtained from the OpenNeuro database. Its accession number is ds000224. (2) Human Connectome Project dataset, n=100 cohort; These data were directly obtained from the HCP website (db.humanconnectome.org)

Field-specific reporting

Please select the one below that is the best fit for your research. If you are not sure, read the appropriate sections before making your selection.

- [x] Life sciences
- [ ] Behavioural & social sciences
- [ ] Ecological, evolutionary & environmental sciences

For a reference copy of the document with all sections, see nature.com/documents/nr-reporting-summary-flat.pdf

Life sciences study design

All studies must disclose on these points even when the disclosure is negative.

Sample size	The presented method is designed to reveal the landscape of brain dynamics in individual participants. It doesn't rely on group statistics for revealing the landscape. Thus, no sample size calculation was required. We selected all available participants from the Midnight Scan Club dataset. For the validation dataset, all available participants in the n=100 cohort of unrelated individuals from Human Connectome Project were used.
Data exclusions	For both datasets (MSC and HCP), all usable data were included in the analyses. Scans were excluded only if the data were corrupted due to excessive head movement artifacts. Please see Methods section 4.2 for details.
Replication	Two replication strategies were used: (1) Within dataset replication, using split-half data validation procedure was performed to replicate results obtained on one half of the MSC data to the next (i.e., odd vs. even session replication); (2) Across dataset replication was performed to replicate results obtained in the MSC data into another dataset from the HCP cohort. All attempts at replication were successful.
Randomization	n/a [no group difference analysis performed]
Blinding	n/a [no group difference analysis performed]

Reporting for specific materials, systems and methods

We require information from authors about some types of materials, experimental systems and methods used in many studies. Here, indicate whether each material, system or method listed is relevant to your study. If you are not sure if a list item applies to your research, read the appropriate section before selecting a response.

Materials & experimental systems

n/a	Involved in the study	
x		Antibodies
x		Eukaryotic cell lines
x		Palaeontology and archaeology
x		Animals and other organisms
	x	Human research participants
x		Clinical data
x		Dual use research of concern

Methods

n/a	Involved in the study	
x		ChIP-seq
x		Flow cytometry
	x	MRI-based neuroimaging

Human research participants

Policy information about studies involving human research participants

Population characteristics	Two already collected datasets were used. (1) Midnight scan club (MSC) data: These data were collected from ten healthy, right-handed, young adult subjects (5 females; age: 24-34). Informed consent was obtained from all participants. The study was approved by the Washington University School of Medicine Human Studies Committee and Institutional Review Board. These data were obtained from the OpenNeuro database. Its accession number is ds000224. (2) Human Connectome Project (HCP) n=100 unrelated cohort: We gathered these data from the Human Connectome Project database. We specifically chose the n=100 unrelated cohort (54 females, mean age=29.1±3.7 years). This cohort of subjects ensures that the participants are not family relatives. As per the HCP protocol guidelines, all participants gave written informed consent for data collection. The HCP scanning protocol was approved by the local Institutional Review Board at Washington University in St. Louis. All experiments were performed in accordance with relevant guidelines and regulations.
Recruitment	n/a [we used already collected data for secondary analysis]
Ethics oversight	For MSC dataset: the Washington University School of Medicine Human Studies Committee and Institutional Review Board. For HCP dataset: Institutional Review Board at Washington University in St. Louis.

Note that full information on the approval of the study protocol must also be provided in the manuscript.

Magnetic resonance imaging

Experimental design

Design type	Resting-state fMRI
Design specifications	For the MSC dataset: Each participant went through 10 sessions of data collection for rsfMRI data, where each session included collection of thirty contiguous minutes of resting state fMRI data, in which subjects visually fixated on a white crosshair presented against a black background.<br><br>For the HCP dataset: A total of 4 resting state fMRI runs were acquired from each participant, where each run was approximately 15 min long. The resting-state fMRI runs (HCP filenames: rfMRI_REST1 and rfMRI_REST2) were acquired in separate sessions on two different days, with two different acquisitions (left to right or LR and right to left or RL) per day
Behavioral performance measures	n/a (resting state scans)

Acquisition

Imaging type(s)	Functional
Field strength	3T
Sequence & imaging parameters	For MSC dataset: Across all sessions, each subject was scanned for 300 total minutes during the resting state. All functional imaging was performed using a gradient-echo EPI sequence (TR = 2.2 s, TE = 27 ms, flip angle = 90, voxel size = 4 mm x 4 mm x 4 mm, 36 slices).<br><br>For HCP dataset: The fMRI data were acquired using whole-brain EPI sequences, with a 32-channel head coil on a modified 3 T Siemens Skyra. The acquisition parameters included were as follows: TR = 720 ms, TE = 33.1 ms, Voxel size = 2.0 mm isotropic. A multi-band acceleration factor of 8 was used to increase temporal resolution.
Area of acquisition	Whole brain

Diffusion MRI ☐ Used ☒ Not used

Preprocessing

Preprocessing software	fMRIprep v.1.5.9
Normalization	Volume-based spatial normalization to two standard spaces (MNI152NLin6Asym, MNI152NLin2009cAsym) was performed through nonlinear registration with antsRegistration (ANTs 2.2.0), using brain-extracted versions of both T1w reference and the T1w template.
Normalization template	MNI152NLin6Asym, MNI152NLin2009cAsym
Noise and artifact removal	Denoising was performed in both datasets using nuisance regression of motion parameters derived by retrospective motion correction, the global signal averaged over the brain, and signal from the WM and CSF
Volume censoring	Frame censuring was performed using a threshold based on Framewise displacement (FD) > 0.2 mm

Statistical modeling & inference

Model type and settings	This work present analysis done at the single participant level, hence no group modeling/statistics were done
Effect(s) tested	Topological and topographical properties of the Mapper-generated graphs were tested using standard statistical methods (e.g., ANOVA)

Specify type of analysis: ☒ Whole brain ☐ ROI-based ☐ Both

Statistic type for inference (See Eklund et al. 2016)	No group maps were generated in this work. However, to evaluate network based statistics nonparameteric spatial mixture modeling based analysis were run
Correction	No group analysis were run. But parameter perturbation and comparison to linear null models was performed to assess properties in the real data (as compared to null generated data)

Models & analysis

n/a	Involved in the study
☒	☐ Functional and/or effective connectivity
☐	☒ Graph analysis
☒	☐ Multivariate modeling or predictive analysis

Graph analysis	Topological properties of the Mapper-generated graph were extracted using the open-source Brain Connectivity Toolbox (https://sites.google.com/site/bctnet/). Properties like degree distribution and centrality were assessed using the BCT toolbox.
